# Genetics of Multiple System Atrophy and Progressive Supranuclear Palsy: A Systemized Review of the Literature

**DOI:** 10.3390/ijms24065281

**Published:** 2023-03-09

**Authors:** Anastasia Bougea

**Affiliations:** 1st Department of Neurology, Medical School, Eginition Hospital, National and Kapodistrian University of Athens, 11528 Athens, Greece; abougea@med.uoa.gr or annita139@yahoo.gr

**Keywords:** multiple system atrophy (MSA), progressive supranuclear palsy (PSP), mutations, synuclein alpha (*SNCA*) polymorphisms

## Abstract

Multiple system atrophy (MSA) and progressive supranuclear palsy (PSP) are uncommon multifactorial atypical Parkinsonian syndromes, expressed by various clinical features. MSA and PSP are commonly considered sporadic neurodegenerative disorders; however, our understanding is improving of their genetic framework. The purpose of this study was to critically review the genetics of MSA and PSP and their involvement in the pathogenesis. A systemized literature search of PubMed and MEDLINE was performed up to 1 January 2023. Narrative synthesis of the results was undertaken. In total, 43 studies were analyzed. Although familial MSA cases have been reported, the hereditary nature could not be demonstrated. COQ2 mutations were involved in familial and sporadic MSA, without being reproduced in various clinical populations. In terms of the genetics of the cohort, synuclein alpha (*SNCA*) polymorphisms were correlated with an elevated likelihood of manifesting MSA in Caucasians, but a causal effect relationship could not be demonstrated. Fifteen MAPT mutations were linked with PSP. Leucine-rich repeat kinase 2 (*LRRK2*) is an infrequent monogenic mutation of PSP. Dynactin subunit 1 (*DCTN1*) mutations may imitate the PSP phenotype. GWAS have noted many risk loci of PSP *(STX6* and *EIF2AK3*), suggesting pathogenetic mechanisms related to PSP. Despite the limited evidence, it seems that genetics influence the susceptibility to MSA and PSP. *MAPT* mutations result in the MSA and PSP pathologies. Further studies are crucial to elucidate the pathogeneses of MSA and PSP, which will support efforts to develop novel drug options.

## 1. Introduction

Progressive supranuclear palsy (PSP) and multiple system atrophy (MSA) are infrequent fatal atypical Parkinsonian syndromes (APS) with miscellaneous phenotypes that impede prompt diagnosis, i.e., about 24% of patients are wrongly diagnosed with idiopathic Parkinson’s disease (iPD) or other neurological conditions [1]. PSP is a disabling tauopathy with an estimated annual prevalence of 5–7 per 100,000 persons and an annual incidence of between 0.9 and 2.6 per 100,000 persons, defined by supranuclear palsy, postural disequilibrium with falls, and executive decline in early stages of the disease. The diagnosis of Parkinsonian PSP-P, the most common PSP variant, is made if the patient has akinetic-rigid, predominantly axial, and levodopa-resistant Parkinsonism (A2) or tremor-dominant and/or asymmetric and/or partially-levodopa-responsive Parkinsonism (A3) [2]. Setting it apart from the two other α-synucleinopathies, dementia with Lewy bodies (DLB) and PD, MSA is defined by cerebellar, extrapyramidal, and pyramidal signs, dysautonomia, and a relatively intact cognitive status. The prevalence of MSA was calculated as 3.4–4.9 persons per 100,000 and the average incidence at 0.6–0.7 cases per 100,000 person-years. Conventionally, MSA is subcategorized into a Parkinsonian MSA-P with prominent, usually symmetrical Parkinsonism, and MSA-C with prominent cerebellar signs [3]. MSA-C has been noted to be more frequent than MSA-P in Japanese people, though the same was not found in Europe and North America [3].

Although PSP and MSA are generally considered sporadic syndromes, familial PSP [4,5,6], MSA [7,8,9,10], two pedigrees with PSP [11,12] or MSA-like presentations [13], and a model autosomal dominant transmission have been suggested [4,5]. In a large retrospective study of 375 autopsy-proven PSP patients, after eliminating those with a MAPTmutation, 18.2% of the remaining sample comprised sporadic PSP patients, and15% of the sample reported having a family background of PSP, Parkinsonism, or dementia [14]. Familial aggregation in PSP was supported by patients with PSP reporting a larger number of family members with Parkinsonism or dementia compare to controls [15]. Meanwhile, genome-wide association studies (GWAS) in large cohorts have revealed several loci significantly linked with PSP [16] and MSA [17], indicating a need for future genetic studies on PSP and MSA.

A previous review concluded that there are no susceptibility genes for MSA [18], while another review suggested the vulnerability of certain genes to PSP pathology (such as *MAPT*) [19]. This review briefly presents the genes that are likely linked with MSA (*SNCA*, *COQ2*, *MAPT*, *GBA1*, *LRRK2*, and *C9orf72*) and those associated with PSP (*MAPT*, *LRRK2*, *DCTN1*, *NPC1*, *PARK2*, *TARDBP*, *GRNTBK1*, and *C9orf72*) highlighting the overlapping genetic pathways of MSA and PSP and suggesting future research directions.

## 2. Materials and Methods

### 2.1. Study Design

Although the scope of this study did not extend to performing a systematic review, I applied the basic principles of a systematic review, but restricted to published peer-reviewed articles and a narrative analysis [20]. I searched the PubMed and MEDLINE databases for peer-reviewed articles focusing on the role of genetics in MSA and PSP and their effect on disease pathogenesis, including articles written in the English language, with no time restrictions. The search was conducted between March 2022 and August 2022. I used the terms “progressive supranuclear palsy”, “multiple system atrophy”, “*GBA1*”, “*COQ2*”, “*MAPT*”, “*SNCA*”, “*LRRK2*”, “*DCTN1*”, “*NPC1*”, “*PARK2”*, “*TARDBP*”, “*GRN*”, “*TBK1*”, “*C9orf72*”, and “GWAS” in various combinations. Relevant articles were screened based on the title and abstract, then read in full. Studies that did not present results were ruled out. Through the snowballing process, I also screened the bibliography of each selected paper for potential additional studies to source the majority of the recent key evidence [21].

### 2.2. Inclusion Criteria

The inclusion criteria were as follows: human, animal, and cell-culture studies published in English.

### 2.3. Exclusion Criteria

The exclusion criteria were as follows: (1) Parkinsonian syndromes beside MSA and PSP, and (2) reviews, letters, editorials, abstracts, conference proceedings, and theses.

## 3. Results

**Figure 1:**
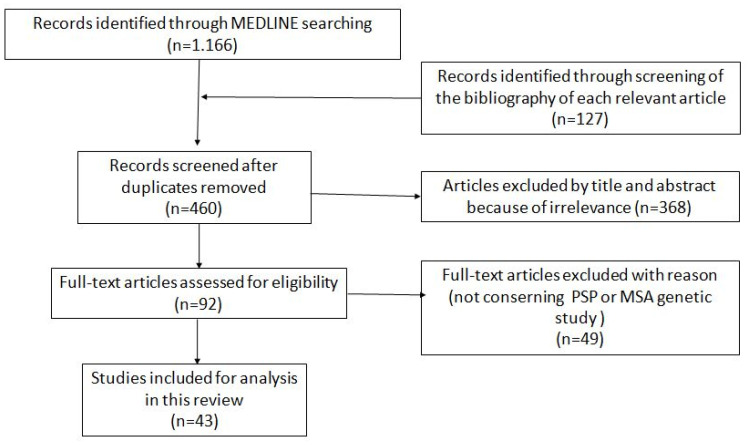
Flowchart of the study selection.

From 1166 studies, after removing 833 duplicates and certain studies ruled out by the exclusion criteria, 132 reports were eligible for review according to the inclusion criteria. Finally, after a full-text review, 43 studies concerning PSP and MSA mutations were selected for analysis; Figure 1 presents a flowchart of the study selection.

Table 1 summarizes the MSA and PSP gene mutations that were confirmed in patient cohorts. Narrative synthesis of the results was then undertaken, as will be described in the following sections.

### 3.1. SNCA—α-Synuclein and MSA

α-Synuclein (encoded by the gene SNCA, in which missense or multiplication mutations cause autosomal dominant PD) is a key element of Lewy bodies in PD [22]. In MSA, however, aggregated abnormal α-synuclein constitutes a major element of GCI in oligodendrocytes [23]. Aggregation of α-synuclein diminishes the capacity of oligodendrocytes to ensure axonal integrity, terminal degeneration, and ultimately, cell death.

α-Synuclein mutations (*A30P*, *E46K*, *H50Q*, *G51D*, and *A53T*) are associated with PD [24,25]. However, causality has not been determined between *SNCA* mutations and MSA, and the relationship between variant α-synuclein and MSA remains unverified. Genetic analyses have studied the inheritance of *SNCA* variants in MSA families [26,27,28]. The *SNCA G51D* variant possesses histopathological features of both MSA and PD [22,26]. Even though G51D was not confirmed only in cases with autopsy-proven pathology, it may be linked both with MSA and PD. Equivalently family members of Finnish origin with the*SNCA*A53E variant shared typical MSA and PD histopathological signatures [27]. As the disease advances, an important accumulation of α-synuclein in non-myelinating oligodendroglial cells occurs only in PD—at this late phase, it is hard to demonstrate a causal association between *SNCA* and MSA—highlighting these mutations’ relevance for PD but not MSA onset [28].

The connection of *SNCA* single-nucleotide polymorphisms (SNPs) with MSA was examined. Four SNPs (rs3857059, rs11931074, rs3822086, and rs3775444) were linked with high risk of MSA in European populations [29,30] but not in a Chinese population [31]. Notably, there was a significant link between rs3822086 and MSA-C.

### 3.2. COQ2—Coenzyme Q2 Polyprenyltransferaseand MSA

*COQ2* produces the enzyme coenzyme-Q2-polyprenyltransferase in the biosynthesis of coenzyme Q_10_, which serves to transport electrons from complexes I and II to complex III [32]. Deficits in coenzyme Q_10_impair oxidative phosphorylation and enhance the vulnerability of cells to harm by free radicals [32]. In a recent meta-analysis, V393A was shown to be a susceptibility variant instead of causative for MSA (specifically, MSA-C) in East Asian cohorts [33]. Meanwhile, other studies did not detect any pathogenic COQ2 variants in MSA [34,35,36], or some detected *COQ2* variants aside from V393A (e.g., L25 V, M128 V, R173H, L402F, A32A, and N386I) in different cohorts [37,38,39].

Reduced plasma coenzyme Q_10_levels in MSA compared to healthy subjects support a pathogenetic role of *COQ2*in MSA [37]. Kasai et al. [40] found an important reduction in serum CoQ10 concentrations in 18 MSA patients compared to controls. Total circulatory cholesterol represents a blood lipoprotein biomarker that delivers CoQ10 in the circulation. Du et al. [41] reported a significant reduction in plasma CoQ10 levels of 40 MSA patients compared to controls. Compta et al. [42] reported decreased CSF CoQ10 concentrations in 20 MSA patients. Moreover, lower coenzyme Q_10_levels were associated with elevated mitochondrial disorder and oxidative stress in the cerebellum of postmortem MSA patients compared to controls [43]. Interestingly, the COQ2 phenotype was not specified for these MSA cases. Overall, current evidence cannot fully rule out a link between specific*COQ2*variants and MSA in ethnicities, a downlink that could be susceptible to MSA. More genetic studies are needed to analyze how *COQ2*assiststhe MSA pathogenesis and resolve the dissimilarities between various ethnic groups.

### 3.3. MAPT—Microtubule-Associated Protein Tauand MSA

Tau, a microtubule-associated protein, is abundant in the axons of neurons with a crucial role in controlling the dynamic behavior and spatial organization of microtubules [44]. In the phosphorylated stage, aggregated Tau disconnects from the microtubules, leading to microtubule inconstancy and demolition [45]. Tau is the gold standard of Taupathies (Alzheimer’s disease (AD), frontotemporal dementia (FTD), corticobasal degeneration (CBD), PSP, Pick’s disease (PiD)) [45]. Despite the existence of Tau in GCI [46], the implication of Tau for the MSA pathogenesis and development is debated.

The MAPT locus consists of two main haplotypes, H1 and H2. The H1c haplotype contributes to PD and PSP [47], while The H2 haplotype rs870723-G allele is correlated with a low likelihood of delayed-onset AD [48]. The H1 haplotype was proven to be a risk factor for 61 autopsy-proven MSA patients [49]. In contrast, however, in the same study, H2 and H1E were not identified as risk factors for 127 autopsy-proven MSA patients. H1x and H1J, meanwhile, have been shown to influence MSA onset [50]. Notably, these results suggest a larger effect size in MSA-C than MSA-P for H2. A further point to note is that six human isoforms of Tau exist, but their role in MSA remains obscure.

### 3.4. GBA1—Beta-Glucocerebrosidaseand MSA

*GBA1* produces an enzyme beta-glucocerebrosidase, which cleaves glucocerebroside from glycolipid glucosylceramide. Mutations in*GBA1*provoke aggregation of glucocerebroside in various tissues, and the onset of Gaucher’s disease (GD) [51]. *GBA1*mutations are also frequent genetic factors for PD and DLB [47]. Regarding the *GBA1*-linked mutations in MSA, the carrier frequency was 1.75% across Japanese, European, and North American groups [52]. *GBA1*mutations were found in MSA-C [52]. *GBA1*mutations—N370S, T369 M, and R496—were detected in four MSA patients among 17 autopsy-confirmed cases of MSA [53]. No link was found between *GBA1*mutations and 108 MSA neuropathologically proven MSA patients [54]. Moreover, 54 Chinese MSA patients were negative for the*GBA1*L444P mutation [55]. Additionally, no causal link was proven with 54 GBA genes in 375 MSA cases [56]. Importantly, GBA mutations do not appear to be involved in the predisposition to MSA (as concluded from 167 autopsy-proven cases) [57]. This may indicate diverse involvement of GBA-mediated lysosomal damage in distinct forms of Parkinsonism. Consequently, despite some initial findings, much remains unknown about whether *GBA1* is linked to MSA (Table 2).

### 3.5. LRRK2—Leucine-Rich Repeat Kinase 2 and MSA

LRRK2encodes an enzyme with a complicated interaction eventually controlling catalytic GTPase and kinase functions [58]. This is critical as three LRRK2mutations in the GTPase domain (R1441C, R1441G, and R1441H) and two in the kinase domain (G2019S and I2020T) are linked with an elevated risk of PD. The most frequent, the G2019S mutation, occurred for 3–10% of familial PD and 1–8% of sporadic PD in European groups [59]. *LRRK2* mutations, such as G2019S, have also been investigated in MSA but, so far, no association between *LRRK2*mutations and MSA has been confirmed [60]. Furthermore, no correlations were demonstrated between *LRRK2*variants (R1628P and G2385R) and 318 MSA patients in the Han Chinese population [61]. A study of American and British cohorts totaling177 MSA autopsy-proven cases showed that M2397 T was a protective haplotype for MSA [62] and, in a recent MSA case report, the G2019S*LRRK2*mutation was confirmed after autopsy-proven diagnosis [63], but larger studies are needed to confirm if*LRRK2*is associated with MSA.

### 3.6. C9orf72—Chromosome 9 Open Reading Frame and MSA

Expansion of the GGGGCC repeat sequence in the*C9orf72*gene is the most frequent risk of both amyotrophic lateral sclerosis and FTD [64]. Earlier reports did not identify a causal association between *C9orf72* and MSA [65,66,67]. However, a case of MSA (according to the only clinical diagnostic criteria for MSA) was reported in a family with ALS and *C9orf72* hexanucleotide pathological expansions (>40) [68]. Furthermore, intermediate repeat expansions in *C9orf72* were revealed in an MSA group from Sardinia, suggesting a high likelihood of MSA [69], and two heterozygous patients were identified with >30 repeats among a cohort of 100 Italian patients [70]. More recently, the first autopsy-proven patient with ALS-*C9orf72* and MSA was reported [71]. These findings highlight the intricate but unexplored role of this gene in the multifariousness of MSA.

### 3.7. MAPT Mutations and PSP

PSP patients with MAPT mutations appear at a wide range of frequencies, between 0.6% and 14.3% [11,12,72]. Fifteen MAPT mutations have been published with PSP-fulfilling clinical or pathological diagnostic criteria (Table 3) [5,72,73,74,75,76,77,78,79,80,81,82,83,84,85,86,87,88].

Beside the R5L mutation in exon1, the V363A in exon12, the R406W in exon13, and other causative mutations in exon 10 are reported to augment the 4R/3R Tau mRNA ratio. The most frequent MAPT mutation related to PSP was found in codon 279 of 11 patients. p.K298 H299insQ in exon 10 was reported in three familial PSP cases, becoming the first insertion mutation published in MAPT. The onset symptoms of PSP *are not clear-cut*, with an enormous clinical variety of PSP, as well a need for follow-up. Patients with PSP and MAPT mutations had a mean age of onset of 44.8 years, though in two families with the N296 mutation, it occurred at an earlier age. Table 1 presented all patients with MAPT mutations and a family background of Parkinsonism, dementia, or other neurodegenerative diseases, supporting the notion of familial aggregation of PSP, as previously published [73]. Thus, it is crucial to exclude MAPT mutations in the case of a positive family history. Recently, a genome-wide survey of copy number variants found MAPT duplications in two patients with pathologically proven PSP and an early age of disease development [16]. These copy number variants encompassed Tau and other genes within the chromosome 17 haplotype region, strengthening the hypothesis that there is a direct pathogenicity of MAPT in PSP [16].

### 3.8. LRRK2 Mutations in PSP

GWAS did not relate LRRK2 to the PSP risk [89,90,91], but did relate it to disease progression [92]. Five mutations were reported in patients with a PSP syndrome [93,94,95,96,97,98,99,100,101] (Table 4).

A large family cohort with R1441C mutation and multiform pathology included 10 members who were distinguished by Parkinsonism and one with neuropathological PSP-like lesions [93]. The R1441H mutation was reported in a case with the PSP-Parkinsonism phenotype (PSP-P) [94]. The contribution of other genetic causes (MAPT haplotype) remains unexplained. In a separate finding, a different study did not detect R1441 mutations or variants [95]. Meanwhile, G2019S was found in four PSP reports with clinical and neuropathological variability [96,97,100]. Moreover, functional in vitro experiments demonstrated that the G2019S mutation exacerbated kinase function, potentially explaining the presence of Tau or α-Synuclein, through enhanced phosphorylation [102]. The fact that a single G2019S mutation can trigger diverse neuropathological scenarios suggests a possible interplay between mutated LRRK2 protein and other genetic loci [95]. The T2310M mutation was identified in a patient with PSP and 27 other rare non-synonymous variants [98]. A novel “disease damaging” p.A1413T mutation was confirmed in a large case–control study of 1039 PSP and 145 CBD cases by several in silico predictive algorithms [99]. It can be surmised that LRRK2 mutations are rarely associated with PSP despite conflicting outcomes. Larger cohorts are warranted to further explore the LRRK2 mutation mechanisms related to Tauopathy and determine whether a high PSP risk can be explained by novel mutations.

### 3.9. Other Genes and PSP

Dynactin subunit 1 (DCTN1) is part of the dynein–dynactin motor protein complex, which plays a crucial role in microtubule binding and molecular trafficking [103]. DCTN1 mutations were found in rare familial neurodegenerative motor diseases. The molecular background in DCTN1 mutation carriers is a transactive response DNA-binding protein of 43 kDa (TDP-43) proteinopathy [103]. There are three DCTN1 mutations that have been identified in PSP-mimicking syndrome [104,105,106]. The mean age at onset was 56.3 years, featuring prominent Parkinsonism and symmetrical frontal atrophy. DCTN1 mutations are also involved in susceptibility to PSP. However, given the rare reports, the molecular interplay between DCTN1 and Tauopathy is still unknown and awaits confirmation.

So far, little evidence has been published on the PSP phenotype and links to other genes (e.g., NPC1 gene, C9orf72 gene, parkin gene (PARK2), transactivation response element DNA-binding protein gene (TARDBP), progranulin gene (GRN), TANK-binding kinase 1 gene (TBK1) and bassoon gene (BSN)) [19].

### 3.10. Genetic Factors Identified by GWAS

GWAS are powerful tools for identifying risk factors for PSP and molecular pathways related to the PSP pathogenesis [89,90,91,92]. Eukaryotic translation initiation factor 2 alpha kinase 3 (EIF2AK3) reveals the endoplasmic reticulum unfolded protein response (UPR) [107]. Increased activity of EIF2AK3 is related to the Tau pathology in PSP [107]. Moreover, deficiency of PERK because of EIF2AK3 mutations leads to the Tau pathology. Beyond that, the MOBP gene increases the PSP risk by modifying myelin or oligodendrocyte functionality. Previous GWAS revealed links of PSP with the MAPT, MOBP, STX6, and EIF2AK3 genes [89,90,91,92]. One revealed an intronic variant (rs564309), found within the tripartite motif-containing protein 11 (TRIM11) gene in chr. 1q42.13, which robustly affects the clinical picture in PSP [92]. However, despite the technological progress, an enormous degree of pathological, clinical, and genetic heterogeneity remains unresolved.

## 4. Discussion

To date, many reports of familial MSA have been published, without implicating hereditable mutations. COQ2 mutations have been found in familial and sporadic MSA, but without being verified in diverse patient cohorts. Genetic SNCA polymorphisms were detected as risk factors of MSA in a Caucasian cohort, but the researchers were unable to confirm other pathogenic mutations. In other work, 15 MAPT mutations were associated with PSP, while LRRK2 has been identified as an infrequent monogenic risk factor of PSP, and it has been demonstrated that DCTN1 mutations may imitate PSP syndromes. GWAS have found risk loci of PSP (EIF2AK3), suggesting pathogenetic mechanisms related to PSP.

There are significant gaps in our knowledge of the effect of genes (SNCA, MAPT, and COQ2) in MSA. Larger studies are warranted to explain the susceptibility genes that play a role in the MSA pathogenesis and process. A larger GWAS of Caucasian vs. Asian cohorts is needed to precisely uncover ethnic genes. As-yet-undetermined areas of research into MSA genetics also concern genome-wide CNV screening, gene isoform diversity, and the effects of various gene haplotypes. Future research must focus not only on the genes traditionally associated with the neurodegeneration process but also on genes in MSA-related pathologies, specifically those resulting in oligodendroglial and mitochondrial damage.

Several studies demonstrated that MAPT gene mutations provoke monogenic autosomal dominant PSP [5,72,73,74,75,76,77,78,79,80,81,82,83,84,85,86,87,88]. In sporadic reports, genetic breakthroughs verified the function of MAPT as a PSP risk factor, and the H1 MAPT haplotype was linked with PSP, while it was proposed that the H2 haplotype may be protective [89]. In contrast, there is conflicting evidence concerning the role of MAPT mutations and haplotypes in MSA development [50]. Further analyses are needed to confirm the roles of other haplotypes in developing PSP or MSA.

Although GBA mutations have been associated with PD and DLB, there have been negative results regarding the link between GBA and MSA [56,57]. This could be explained by unknown environmental factors that may interfere with the MSA pathology. More prospective studies should access these factors.

An important point revealed by this review is that unique signs at the LRRK2 locus are correlated with the disease course for PSP, but not the PSP risk [87,90]. These results may explain cell-type-specific expression models or variability in the pathology and disease stages and clarify the molecular pathways in PSP, confirming the potential of LRRK2 as a therapeutic agent. Moreover, the pathological pathway from the PSP evolution variant to LRRK2 could be explained by the effect of LINC02555 on the expression or translation of LRRK2 mRNA in specific cells, thus increasing LRRK2 protein titles [105]. However, it remains to be confirmed whether the PSP-linked variant provokes hyperactive LRRK2 kinase and Rab phosphorylation [108]. The identification of diverse patterns of Rab phosphorylation in future research may result in new therapeutic options for PSP.

The prevalence of C9ORF72 expansion in PSP and MSA is challenging to calculate based only on a few case reports and small cohort studies [65,66,67,68,69,70,71,89], compounded by the difficulty of phenotypic classification of complex syndromes with Parkinsonian characteristics. Autopsy studies on such patients often reconsider the initial clinical diagnosis. No studies on C9orf72 in autopsy-proven patients with MSA confirmed the previous clinically determined groups [70,71]. Future prospective studies with numerous autopsied cases of MSA and PSP are needed to resolve this problem.

There are many open questions that need to be addressed by future studies. Very little evidence has been published on the PSP phenotype (NPC1 gene, C9orf72 gene, PARK2, TARDBP, GRN, TBK1, and BSN) [19]. Moreover, MSA and PSP may share the same LLRK2 G2019S mutation, and, given the overlapping phenotypes, there is a strong hypothesis that atypical Parkinsonian syndromes may share common pathological mechanisms. Should we consider neurodegeneration process as a spectrum of pathologies including MSA and PSP? What might be the exact role of genetics within the interplay of other factors such as environmental ones?

Most of the included studies had several limitations, such as limited samples, absence of a control group, and absence of pathologically confirmed cases or copathologies. Indeed, case studies were mainly performed, which are interesting but have significant limitations in terms of extrapolation to all MSA and PSP cases [65,66,67,68,69,70,71,93,94,95,96,97,98,99,100,101]. Furthermore, there were no associations between sex/gender and mutations in the included studies, but many genetic results were influenced by ethnicity or geographical areas, suggesting multiple factors causing MSA-associated gene variants. Notably, confounders such as diverse progression/therapies may have affected the results, and only one time point (i.e., end stage) in the disease was explored. Moreover, the exact role of the interplay between genes and the environment in the pathophysiology of PSP and MSA remains to be determined.

This review also has its limitations. First, given the high diversity of the results, meta-analysis was not conducted. Second, there are few reports of atypical Parkinsonian or dementia syndromes to compare. Third, only the PubMed and MEDLINE databases were searched, though I ensured that all available bibliographies were included.

Finally, this review has certain strengths worth noting: the literature was up-to-date and included the latest evidence, and effective inclusion and exclusion criteria were determined for study selection.

## 5. Conclusions and Future Directions

So far, the genetic studies in MSA have failed to demonstrate genetic causes (SNCA, COQ2, SCAs, expansions, etc.) of accurately diagnosed MSA. Although many gene polymorphisms have been proposed as MSA risk factors, the results have been contradictory due to methodological flaws (e.g., high heterogeneity of the MSA cohorts).

Genetics may be involved in the vulnerability to PSP, and mutations of MAPT may result in the PSP pathology. Today, the link between genes and PSP needs confirmation, which it is important that we work toward since it may support future genetic screening, such as testing for MAPT mutations, e.g., in PSP familial members.

Future genetic analyses with larger cohorts are warranted to identify more novel loci and their roles in the etiopathogeneses of PSP and MSA. Although limited, our knowledge of the genetics of MSA and PSP centers on overlapping pathways such as protein aggregation, impaired intracellular trafficking, and dysfunctional protein degradation. Functional analysis will be important to further explain underlying molecular mechanisms, thus supporting work to develop new diagnostic biomarkers and therapeutic targets for PSP and MSA. Researchers should seek to overcome the high cost barrier of genetic screening on expanded clinical cohorts to examine different genotype–phenotype links and discover unpredictable disease connections.

## Figures and Tables

**Table 1:** Summary of MSA and PSP gene mutations.

GBA-MSA	LRRK2-MSA	MAPT-PSP	LRRK2-PSP
*L444P-A456P-V460V* or *Rec*Nci*I*	*G2019S*	*R5L*	*R1441C*
*N370S, T369M* and *R496H*		*N279K*	
*R262H*			*R1441H*
*L444P*		*L284R*	
*N409S* *L483P*		*S285R*	*G2019S*
		*Δ* *N296*	
			*T2310M*
		*K298_H299insQ*	*A1413T*
			*G2019S*
		*P301L*	
		*G303V*	
		*S305S*	
		*IVS10+3G>A*	
		*IVS10+14C>T*	
		*IVS10+16C>T*	
		*V363A*	
		*R406W*	

GBA, beta-glucocerebrosidase; MAPT, microtubule-associated protein Tau; MSA, multiple atrophy sclerosis; PSP, progressive supranuclear palsy.

**Table 2:** GBA variants causing MSA.

Study	Sample	Mutation Type	Clinical Phenotypes	Family History	Clinical Diagnosis	Neuropathologic Diagnosis
Mitsui et al. [52]	969 MSA (574 Japanese, 223 European, 172 N. American)	L444P-A456P-V460V or Rec*Nci*I	MSA-Parkinsonism and MSA-Cerebellar	Yes	MSA	NA
Sklerov et al. [53]	17 MSA + AD	N370S, T369M, and R496H (4 pts/ 3 Ashkenazi Jewish)	Parkinsonism, orthostasis, urinary symptoms, constipation, erectile dysfunction, RBD	Yes (2 pts) No (2 pts)	MSA	MSA
Segarane et al. [54]	108 MSA + 257 C (British origin)	*R262H*	NA	NA	MSA	MSA
Sun et al. [55]	54 MSA, 109 ET, 657 C (all Chinese)	*L444P*	NA	No	MSA	NA
Wernick et al. [56]	167 MSA + 834 C	*N409S* *L483P*	NA	NA	MSA	MSA

AD, Alzheimer’s disease; C, controls; ET, essential tremor; MSA, multiple atrophy sclerosis; NA, not available; RBD, rapid eye movement (REM) sleep behavior disorder.

**Table 3:** MAPT mutations causing PSP-like syndrome.

Study	Sample	Mutation Type	Clinical Phenotypes	Family History	Clinical Diagnosis	Neuropathologic Diagnosis
Poorkaj et al. [72]	96 PSP + 4 C	*R5L*	falls, dysarthria, micrographia	No	PSP	PSP
Yasuda et al. [73]		*N279K*	Parkinsonism	Yes	PNLD	PSP-mimicker
Delisle et al. [74]		*N279K*	apathy, memory disorder, Parkinsonism	Yes	FTDP-17	NA
			indifference, attention disturbances	Yes	FTDP-17	NA
Soliveri et al. [75]		*N279K*	personality and behavior changes	Yes	PSP	NA
Ogaki et al. [77]		*N279K*	Parkinsonism, micrographia	Yes	PSP	PSP-mimicker
			oscillopsia, shuffling gait, bradykinesia	Yes	PSP	NA
Ogaki et al. [76]		*N279N*	Parkinsonism	Yes	PSP	NA
Rohrer et al. [78]		*L284R*	falls, personality changes	Yes	PSP	NA
Ogaki et al. [76]		*S285R*	speech and breathing disorder	No	PSP	NA
Fujioka et al. [5]		*S285R*	dystonia and supranuclear gaze palsy	Yes	PSP	PSP-AD
			bradykinesia	Yes	PSP	PSP
Pastor et al. [79]		*Δ*N296	speech and memory disorder	Yes	Atypical PSP	NA
Rossi et al. [80]		*Δ*N296	falls, antecollis, dysarthria	Yes	PSP-mimicker	NA
Nakayama et al. [81]		*K298_H299insQ*	neck stiffness, postural instability	Yes	PSP	NA
			gait difficulty, cognitive dysfunction	NA	NA	NA
Bird et al. [82]		*P301L*	tremor, speech impairment	Yes	APD	PSP-mimicker
Kaat et al. [15]		*P301L*	NA	Yes	PSP	NA
Ros et al. [83]		*G303V*	Parkinsonism, falls, micrographia, dysarthria, ocular motor damage	Yes	PSP	PSP
Stanford et al. [84]		*S305S*	dystonia, dysarthria, falls, bradykinesia	Yes	PSP	PSP
Spina et al. [85]		*IVS10+3G>A*	dizziness, neck rigidity	Yes	Atypical PSP	PSP-mimicker
Omoto et al. [86]		*IVS10+14C>T*	clumsiness, tremor, apathy	Yes	Perry syndrome	PSP-mimicker
Morris et al. [87]		*IVS10+16C>T*	fatigue, micrographia, withdrawal	Yes	PSP	Tauopathy
Rossi et al. [88]		*V363A*	diplopia, falls, bradykinesia	Yes	PSP	NA
Ygland et al. [12]		*R406W*	dyscalculia, social isolation, apathy	Yes	AD	PSP-mimicker

No, number; NA, not available; PSP, progressive supranuclear palsy; PNLD, pallido-nigro-luysian degeneration; FTDP-17, frontotemporal dementia and Parkinsonism linked to chromosome 17; AD, Alzheimer’s disease; PSP-AD, PSP with concomitant AD. “PSP-mimicker” pathology, although present, does not fulfill the pathological criteria of PSP.

**Table 4:** LRRK2mutations of PSP-mimicker phenotypes.

Study	Sample	Mutation Type	Clinical Phenotypes	Family History	MAPT Haplotypes	Clinical Diagnosis	Neuropathologic Diagnosis
Zimprich et al. [93] Wszolek et al. [94]	Family pedigree (22 PD/190 members)	R1441C (1Fpt)	Parkinsonism, supranuclear gaze palsy	Yes	H1/H1	PD	SN neuronal loss + PSP-like changes
Spanaki et al. [95]	266 PD + 13 PSP	R1441H (1Fpt)	Parkinsonism, bulbar impairment dysfunction, major cognitive disorder	Yes	NA	PD	NA
Rajput et al. [97]	85 LBD + 16 PSP + 16 ET + 11 MSA + 4 CBD + 1 PiD + 22 C	G2019S (1Mpt)	Parkinsonism	Yes	H1/H1	PD	PSP + early-stage AD
Ruffmann et al. [98]	1 PD	G2019S (1Mpt)	tremor	No	H1/H1	PSP	PSP + early-stage AD
Trinh et al. [100]	948 PD + 189 (PSP + MSA + DLB) + HC	T2310M	NA	No	NA	PSP	NA
Sanchez-Contreras et al. [101]	1039 PSP + 145 CBD + 1790 C	A1413T (1Mpt)	Parkinsonism, memory disorder, postural instability, eyelid apraxia	No	H1/H1	PSP	PSP + AGD
		G2019S (1Fpt)	Bulbar dysfunction, tremor, retrocolitis	No	H1/H1	PSP	PSP + early-stage AD
Blauwendraat et al. [99]	624 AD + 216 LBD + 27 AD/ALS-FTD + 26 (AD + AP) + 40 complex cases + 50 PD + 49 LBD + 84 ALS-FTD + 13 MSA + 37 PSP + 7 CBD + 9 rare syndromes + 47 unclassified + 14 HS	G2019S (1Fpt)	NA	Yes	NA	PD	PSP + AD

AD, Alzheimer’s disease, ALS-FTD, amyotrophic lateral sclerosis-frontotemporal dementia; AP, atypical Parkinsonism; CBD, corticobasal degeneration; DLB, dementia with Lewy bodies; ET, essential tremor; HC, healthy controls; F, female; LBD, Lewy body diseases; Mpt, male patient; NA, not available; PiD, Pick’s disease; PSP, progressive supranuclear palsy; PSP+AD, PSP with concomitant AD. “PSP-mimicker” pathology, although present, does not fulfill the pathological criteria of PSP.

## Data Availability

The data used to support the findings of this study are available from the corresponding author upon request.
